# Genome Sequence of *Megasphaera cerevisiae* NSB1, a Bacterium Isolated from a Canning Line and Able To Grow in Beer with High Alcohol Content

**DOI:** 10.1128/genomeA.01686-16

**Published:** 2017-02-23

**Authors:** Jordyn Bergsveinson, Euan Thomson, Derek Jacoby, Yvonne Coady, Barry Ziola

**Affiliations:** a Department of Pathology and Laboratory Medicine, College of Medicine, University of Saskatchewan, Saskatoon, Saskatchewan, Canada; b Phillips Brewing and Malting Co., Victoria, British Columbia, Canada; c Department of Computing Science, Faculty of Engineering, University of Victoria, Victoria, British Columbia, Canada

## Abstract

The genome sequence of the brewery isolate *Megasphaera cerevisiae* NSB1 was determined. Strain NSB1 tolerates 5% (vol/vol) alcohol, which is higher than previously reported for *M. cerevisiae*. The NSB1 genome will help elucidate genetics required for alcohol tolerance and niche adaptation of this Gram-negative beer-spoilage bacterium.

## GENOME ANNOUNCEMENT

Beer spoilage by the anaerobic Gram-negative bacterium *Megasphaera cerevisiae* is severe, as it produces strong off-flavors and aromas such as acetoin and butyric, acetic, caproic, isovaleric, and caproic acids (1). *M. cerevisiae* contamination is reportedly limited to beers with low alcohol content (<3.5% wt/vol) (2). However, *M. cerevisiae* NSB1, as isolated from a canning line, is able to grow in and spoil hopped ale containing 5% (vol/vol) ethanol with 45 international bitterness units and pH 4.55. *M. cerevisiae* NSB1 was propagated on agar plates of WL Differential media (HiMedia Laboratories, Mumbai, India), containing 4 mg/L cycloheximide and 0.5 g/L cysteine hydrochloride with anaerobic incubation at 30°C. Microbial mass was suspended in MicroBead Solution of a MoBio UltraClean DNA extraction kit (Mo Bio Laboratories, Carlsbad, CA, USA). DNA extraction proceeded according to the manufacturer’s instructions, including an optional heating step of 70°C for 10 min prior to bead beating to increase cell lysis efficiency.

Sequencing was performed by Illumina MiSeq (paired-end 2 × 300 bp; at Contango Strategies Ltd., Saskatoon, SK, Canada). Reads were assembled by SPAdes-3.9.0-Darwin (3), using the “–careful” flag to minimize the number of mismatches. Assembly output was filtered to remove scaffolds <450 bp and coverage scores <2, producing 163 contigs with 40× coverage and an *N*_50_ length of 48,664. The draft genome sequence is 3,015,877 bp long, with a 45% G+C content. Annotation was performed by RAST (Rapid Annotations using Subsystems Technology) version 2.0 (4) via the “RAST*tk*” modular pipeline (5) and PGAP (NCBI Prokaryotic Genome Annotation Pipeline) (6). The PGAP annotation revealed that *M. cerevisiae* NSB1 has 2,827 genes and 2,652 protein-coding sequences, 19 rRNA operon (CDSs), 53 tRNAs, one CRISPR array, and 89 pseudogenes.

*M. cerevisiae* NSB1 contains a complete *suf* operon (involved in iron homeostasis) and the manganese transporter MntH, as described for the genome-sequenced beer-spoilage strain *M. cerevisiae* PAT 1^T^ (DSM 20462^T^) (7). Unlike in PAT 1^T^, the full arginine catabolism operon is not complete in *M. cerevisiae* NSB1. Conversely, *M. cerevisiae* NSB1 contains several CDSs not present in PAT 1^T^, including a cadmium-transporting ATPase, and a complete anaerobic sulfite reductase operon (*asrA, B*, and *C*) involved in iron and sulfite homeostasis. Additionally, several transcripts related to capsular and extracellular polysaccharides metabolism are exclusive to *M. cerevisiae* NSB1.

*M. cerevisiae* NSB1 also contains numerous transcripts related to lactate utilization (l-lactate dehydrogenase and l-lactate permease), which are involved in producing acetic, butyric, and hexanoic acid in *M. elsdenii* (8). Glycerol kinase and glycerol dehydratase pathways, responsible for glycerol degradation, are also present in *M. cerevisiae* NSB1, likely further contributing to beer off-flavor through production of highly volatile acid and aroma compounds, as has been found for the Gram-positive beer-spoilage organism *Pediococcus pentosaceus* CAg (9). A large assortment of multidrug efflux pump genes are present, as are multiple copies of alcohol dehydrogenase genes, which all may contribute to the export of solvent/ethanol, thus explaining the unusual high-alcohol tolerance of *M. cerevisiae* NSB1. The genetics of *M. cerevisiae* NSB1 are worthy of further exploration from the perspective of understanding the beer-spoilage capacity of *M. cerevisiae*.

### Accession number(s).

The *M. cerevisiae* NSB1 genome was deposited in GenBank under the accession number MQNM00000000.
